# The Application of S-Substituted Pteridine for CCl_4_-Induced Acute Hepatitis Treatment in Rats

**DOI:** 10.3390/biomedicines13061276

**Published:** 2025-05-22

**Authors:** Natalia Lohvinenko, Volodymyr Shvets, Oleksii Antypenko, Oleksii Voskoboinik, Andrii Bozhkov, Hanna Maslak, Valentyn Oksenych, Oleksandr Kamyshnyi, Sergiy Okovytyy, Serhii Kovalenko

**Affiliations:** 1 Department of Physiology, Immunology, Biochemistry with a Course in Civil Defense and Medicine, Zaporizhzhia National University, 69063 Zaporizhzhia, Ukraine; 2 Department of Biochemistry, Zaporizhzhia State Medical and Pharmaceutical University, 69000 Zaporizhzhia, Ukraine; 3 Department of Pharmaceutical, Organic and Bioorganic Chemistry, Zaporizhzhia State Medical and Pharmaceutical University, 69000 Zaporizhzhia, Ukraine; 4 Department of Composite Materials, Chemistry and Technologies, National University «Zaporizhzhia Polytechnic», 69063 Zaporizhzhia, Ukraine; 5 Research Institute of Biology, V. N. Karazin Kharkov National University, 61022 Kharkov, Ukraine; 6 Department of Biochemistry and Medical Chemistry, Dnipro State Medical University, 49044 Dnipro, Ukraine; 7 Broegelmann Research Laboratory, Department of Clinical Science, University of Bergen, 5020 Bergen, Norway; 8 Department of Microbiology, Virology, and Immunology, I. Horbachevsky Ternopil National Medical University, 46001 Ternopil, Ukraine; 9 Institute of Chemistry and Geology, Oles Honchar Dnipro National University, 49000 Dnipro, Ukraine

**Keywords:** acute hepatitis, S-substituted pteridine, Lipocalin-2, Nrf2, hepatoprotectors, antioxidants, oxidative stress

## Abstract

**Background/Objectives:** Liver disease is one of the most common medical problems in the world. The pharmacological correction of these pathologies includes the use of drugs with antioxidant and hepatoprotective action, among which there are natural and synthetic sulfur-containing compounds. However, many of these drugs have side effects, and their application does not always correspond to approaches in evidence-based medicine. Therefore, today the urgent problem is the search for new effective substances with high metabolitotropic properties and high safety criteria. The aim of this work was an in-depth study of the hepatoprotective and antioxidant action of a new investigational pteridine-containing “lead-compound” (DCTP) under conditions of experimental tetrachloromethane hepatitis in rats in comparison with the reference drug “Thiotriazoline”. **Methods:** The hepatoprotective effect of the compound was studied using a model of acute tetrachloromethane (CCl_4_) hepatitis in adult male Wistar rats. The levels of biochemical liver damage markers were estimated with spectrophotometric methods. Histological and immunohistochemical methods were used for the determination of hepatocyte damage. The statistical processing of data was performed using the nonparametric Wilcoxon–Mann–Whitney method. **Results:** The results of the studies showed that DCTP was superior to the reference drug Thiotriazoline in terms of its effect on the levels of AST, DC, Schiff bases, and carbonylated proteins, which are markers of oxidative (Nrf2) and inflammatory (Lipocalin-2) stress, as well as its effect on animal survival. The results were confirmed by histological examination data, which showed regeneration of the hepatocyte membrane structure; a reduction in infiltrative, destructive, and inflammatory process in the liver; a reduction in the cytolytic process; stabilization; and an increase in the functional activity of the liver due to the administration of the study drug. The pharmacological effects of the studied compound (DCTP) are probably associated with its structural similarity to tetrahydrofolic acid, which is an integral component of oxidation–reduction processes and a participant in the biosynthesis of nitrogenous bases of nucleotides and amino acids. The obtained data show the antioxidant and hepatoprotective properties of the studied “lead-compound” from the pteridinethione group (DCTP). **Conclusions:** It was shown that the studied substance DCTP significantly reduces acute hepatotoxic effects caused by CCl_4_, as evidenced by the decrease in the level of lipid peroxidation and prooxidant markers, the normalization of liver biochemical markers, the regeneration of the liver architecture, the limitation of inflammatory effects, the decrease in Nrf2 and Lipocalin-2 markers, and the induction of liver antioxidant enzymes.

## 1. Introduction

Liver pathologies of various etiologies are becoming increasingly common in all countries [1]. This can be explained by the fact that liver diseases are induced by an extremely wide range of factors, from viruses to chemicals, drugs [2], and even food and beverages [3]. The problem is that liver diseases go through successive stages in their development—hepatitis, fibrosis, cirrhosis, and/or hepatocellular cancer [4]—and this process can take several years. At the initial stages, it is almost painless. Treatment is very ineffective when its detection occurs at later stages, when the pathology becomes chronic. In this regard, the search for ways to prevent and treat liver pathologies and the development of methods for testing new substances with hepatotropic action remain relevant.

As is known, one of the primary responses to liver dysfunction is a shift in the balance of the redox system towards an increase in prooxidants and the development of oxidative stress [5]. It is important to consider that changes in the redox system’s parameters are accompanied not only, and perhaps not so much, by the formation of negative hepatoxic compounds, but also by the restructuring of regulatory systems in the liver and in the body as a whole [6]. Based on this, we believe that the assessment of the state of the redox system could be a marker in primary tests and the assessment of the action of new hepatopropionic substances.

When selecting hepatoprotective substances, we proceeded from the fact that they should ensure the correction of the redox system. It is known that α-lipoic acid, methionine, S-adenosylmethionine, and L-glutathione can stabilize and inhibit free radical oxidation in hepatocytes [7]. Thus, it has been shown that methionine helps maintain the oxidation–reduction status of cells by forming homocysteine, which is a substrate involved in the transsulfuration of the glutathione pathway [8]. It also acts as a source of sulfur in the synthesis of the critical signaling molecule hydrogen sulfide [9]. S-adenosylmethionine is a universal donor of a methyl group in the methionine cycle in the presence of folates (THF, 5-MTHF, 5,10-MTHF) [10]. We believe that a promising approach to the creation of new effective hepatoprotectors may be the modification of a natural heterocyclic matrix—pteridine. This is supported by data showing that the drug Vesatolimod (an aminopteridine derivative) is undergoing clinical trials for the treatment of acute and chronic forms of hepatitis B [11], and S-substituted pteridines are effective inhibitors of dehydrofolate reductase and free radical scavengers [12], exhibiting antioxidant activity in in vitro experiments and in toxic liver damage [13]. In addition, pteridines, such as tetrahydrofolic acid and tetrahydrobiopterin, act as coenzymes in the synthesis of nucleotides, amino acids, and phospholipids, which are molecules that are necessary for regeneration processes [14].

**Figure 1:**
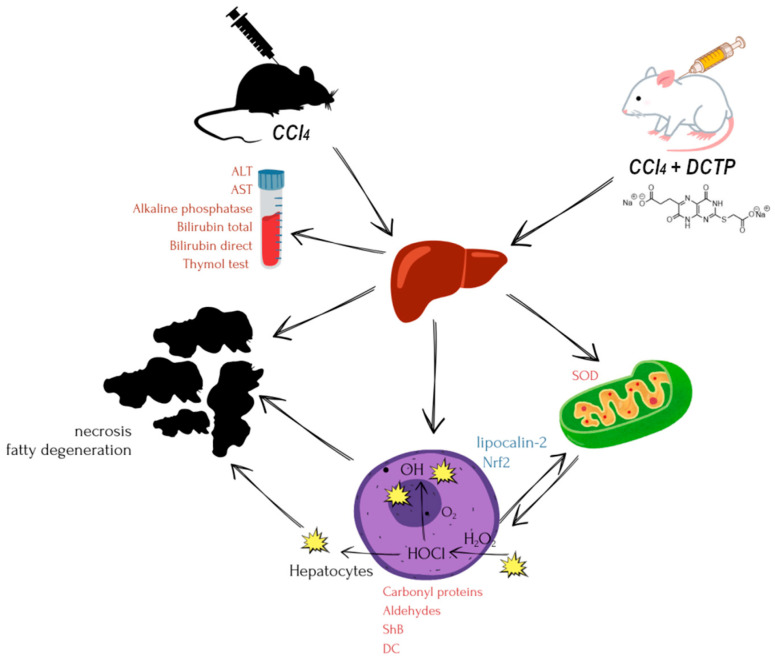
Research design (distribution scheme of materials and research methods).

To test this working hypothesis, an in-depth study of the hepatoprotective and antioxidant effects of the new investigational “lead-compound”, namely disodium 3-(2-((carboxylatomethyl)thio)-4,7-dioxo-3,4,7,8-tetrahydropteridin-6-yl)propanoate (DCTP) [15], was conducted under conditions of experimental tetrachloromethane hepatitis in rats in comparison with the reference drug “Thiotriazoline” (Figure 1). 

## 2. Materials and Methods

The hepatoprotective effect of the compound was studied using a model of acute tetrachloromethane (CCl_4_) hepatitis in 40 adult male Wistar rats (6–8 months) weighing 220–350 g, which were kept under standard vivarium conditions (temperature 20 ± 5 °C, humidity 65 ± 5%). The rats were kept on a standard diet with free access to water and food under conditions of a natural alternation between day and night [16].

Animal care and experimental protocols were carried out in accordance with the requirements of the Council Directive 86/609/EEC of 24 November 1986 on the care and use of laboratory animals, the ethical principles for animal experiments approved by the First National Congress of Ukraine on Bioethics (2001), international agreements and Ukrainian legislation in this area, and Directive 2010/63/EU of the European Parliament, and they were approved by the Ethics Committee [17].

To assess hepatoprotective activity, 40 rats were divided into the following four groups of 10 animals each. Samples obtained from these four groups were used for all biochemical, histological, and histochemical studies.

Group I (control): 10 rats, intact animals, which were intraperitoneally administered with an appropriate volume of 0.9% sodium chloride solution for 14 days.

In rats in groups II-IV, experimental hepatitis was induced by subcutaneous administration of carbon tetrachloride (CCl_4_) at a dose of 0.8 mL/100 g of weight in the form of a 50% oil solution once a day for 2 days [18,19].

Group II (CCl_4_): 10 rats, which were administered only CCl_4_.

Group III (CCl_4_ + TTZ): the 10 rats in this group were given intraperitoneal administration of a 2.5% aqueous solution of Thiotriazoline (TTZ) at a rate of 10 mg/100 g (Arterium, Ukraine, Series: UA/2931/01/02, No.: LSR-0052882 dated 18 February 2015) once a day against the background of CCl_4_ hepatitis from the 1st to the 14th day [20].

Group IV (CCl_4_+ DCTP): the 10 rats in this group were given intraperitoneal administration of an aqueous solution of disodium 3-(2-((carboxylatomethyl)thio)-4,7-dioxo-3,4,7,8-tetrahydropteridin-6-yl)propanoate (DCTP) [15] at a dose of 6 mg/100 g [19] once a day against the background of CCl_4_ hepatitis from the 1st to the 14th day. As indicated by earlier preliminary studies, DCTP is promising experimental drug for the treatment of acute hepatitis [15]. DCTP reliably maintains the protein-synthesizing and detoxifying functions of the liver. The intraperitoneal toxicity of DCTP assessed in mice is 6240 ± 830 mg/kg, thus classifying the compound as practically non-toxic.

The decapitation of animals using ether anesthesia was performed on the 15th day after the termination of the experiment from 9:00 to 11:00.

The liver was divided into three fragments, and the first part was immediately removed and frozen in liquid nitrogen. For the extraction of diene conjugates, frozen liver fragments were homogenized with a mixture of heptane:propan-2-ol (1:1).

The concentration of diene conjugates (DCs) was determined with a ULAB 108UV (Shanghai, China) spectrophotometer at 232 nm using the method of Recknagel and Goshal [21] and expressed as nmol/g liver tissue.

The concentration of Schiff bases in liver extracts was determined with a Hitachi MPF-4 spectrofluorometer (Tokio, Japan) at an adsorption wavelength of 360 nm and an emission wavelength of 430 nm using the method of C.A. Rice-Evans [22] and expressed as nmol/g liver.

Determination of superoxide dismutase (SOD) concentration (EC 1.15.1.1): Liver homogenate was obtained by grinding liver tissue (homogenization) in PBS buffer (pH = 7.4) using a Potter–Elvehjem homogenizer in a 10-fold volume of 0.25 M sucrose. Homogenates were filtered through 2 layers of gauze and centrifuged CL-310b (Warsaw, Poland) at 1000× *g* for 10 min. Then, the supernatant was centrifuged at 10,000× *g* for 20 min. Subsequently, the pellet was suspended in 5 mL of 0.25 M sucrose (pH 7.4) and washed twice at 10,000× g for 20 min. All procedures were performed at 4 °C [23]. The sediment of purified mitochondria was suspended in 1 mL of 0.25 M sucrose.

The concentration of carbonylated proteins in the samples was determined using the method of R.L. Levine [24] as modified by E. Dubinina. This method is based on the interaction of the carbonylated groups and imino groups of oxidized amino acid residues from proteins with 2,4-dinitrophenolhydrazone (2,4-DNPH) to form 2,4-dinitrophenolhydrazones, which have a specific absorption spectrum in the ultraviolet and visible regions of the spectrum. The level of the spontaneous oxidative modification of proteins reflects the amount of carbonyl derivatives of proteins present in the samples (formed in vivo). For this purpose, 0.1 mL of the mitochondrial fraction suspension was mixed with an equal volume of 20% trichloroacetic acid (TCAA). The samples were vigorously shaken, after which 1 mL of 5% TCAA was added to them and they were centrifuged at 3000 rpm for 15 min. The formed supernatant was removed, and the sediment was mixed with 1 mL of 1 M 2,4-dinitrophenylhydrazine solution dissolved in 2 M hydrochloric acid. The samples were mixed and incubated for an hour at room temperature (20–22 °C) and centrifuged at 3000 rpm for 15 min. The supernatant was removed, and the sediment was mixed with 3 mL of ethanol–ethyl ether mixture (1:1) and centrifuged at 3000 rpm for 5 min. The procedure involving sediment washing was repeated three times. The washed sediment was dried at room temperature. After that, it was dissolved in 3 mL of 8 M urea solution. To determine the content of carbonated proteins, the optical density of the resulting solution was measured with a spectrophotometer at 363 nm. The results are presented as nmol per mg of mitochondrial protein.

To obtain serum, blood was collected in glass tubes without anticoagulant. The serum was separated from the blood clot, centrifuged for 15–20 min at 1000× *g*, and then stored at (−20 °C) for the evaluation of biochemical parameters [25].

The study of biochemical parameters was carried out with a semi-automatic open-type biochemical analyzer BioSystems BTS 330 (Barcelona, Spain) with reagent kits manufactured by BioSystems (Barcelona, Spain), and the thymol test was carried out with reagents manufactured by Lachema (Brno, Czech Republic).

The activity of alanine aminotransferase (ALAT) (EC 2.6.1.2) and aspartate aminotransferase (ASAT) (EC 2.6.1.1) was determined in blood serum by the UV-kinetics method (IFCC); the activity of alkaline phosphatase (ALP) (EC 3.1.3.1) was also determined by the UV-kinetics method (IFCC) with 2-amino-2-methyl-1-propanol buffer (AMP) [26]; and the ASAT/ALAT ratio was calculated (De Ritis coefficient) [27].

The total and direct bilirubin levels were determined by the method of Jendrassik and Grof [28].

The thymol test was determined by the intensity of turbidity due to the formation of a globulin–thymol–lipid complex [29].

**Histology:** For histological studies, liver pieces were taken, fixed in 10% neutral formalin solution, and embedded in paraffin. The preparations were stained with hematoxylin and eosin, which were used to study the normal liver structure, as well as the nature and depth of morphological changes after tetrachloromethane intoxication and its complex correction with the studied drugs. Hematoxylin and eosin staining is the most common method of section staining. This method allows you to establish the relationship between the parts of the organ, perfectly revealing all cellular elements and some non-cellular structures. This staining is double: hematoxylin—the main dye—stains the cell nuclei, and eosin—an acidic dye—stains the cell protoplasm and, to a lesser extent, various non-cellular structures. Paraffin sections were passed through a panel of alcohols, stained with Mayer’s hematoxylin and then eosin, and placed under glass for further tissue study. For coloring, ready-made dyes produced by BioPrime (Kronshagen, Germany) were used.

**Immunohistochemistry:** Paraffin blocks with liver tissue were processed as described [30]. The non-specific staining of sections was blocked with 50% FCS and 0.3% Triton X-100 in PBS for 30 min at 37 °C, followed by incubation with peroxidase, avidin, and biotin. Sections were incubated with primary antibodies at 4 °C overnight and then incubated with biotinylated secondary antibodies BioPrime (Kronshagen, Germany) and avidin-conjugated peroxidase BioPrime (Kronshagen, Germany) and processed using a 3,3’-diaminobenzidine substrate BioPrime (Kronshagen, Germany).

The statistical processing of data was performed using the nonparametric Wilcoxon–Mann–Whitney method and the GraphPad Prism 6.0 software package.

## 3. Results

### 3.1. The Hepatoprotective Effect of DCTP on the Model of Toxic Liver Damage Induced by Carbon Tetrachloride

The effect of carbon tetrachloride was manifested by an increase in the activity of ALT by 3.47, AST by 2.08, ALP by 2.55, total bilirubin by 2.06, direct bilirubin by 1.69, and thymol by 5.13 times in the blood serum compared with these indicators in the control group (Table 1). After the administration of DCTP to animals with hepatitis (group IV), the activity of ALT decreased by 59.1% compared with group II (CCl_4_) and corresponded to the activity of the comparison group III (CCl_4_ + TTZ). If we compare the activity of ALT, in animals with hepatitis receiving DCTP (group IV), it remained slightly increased compared to the control animals (Table 1). It should be noted that the administration of TTZ to animals with hepatitis (group III) caused a similar effect, as did DCTP (Table 1).

**Figure 2:**
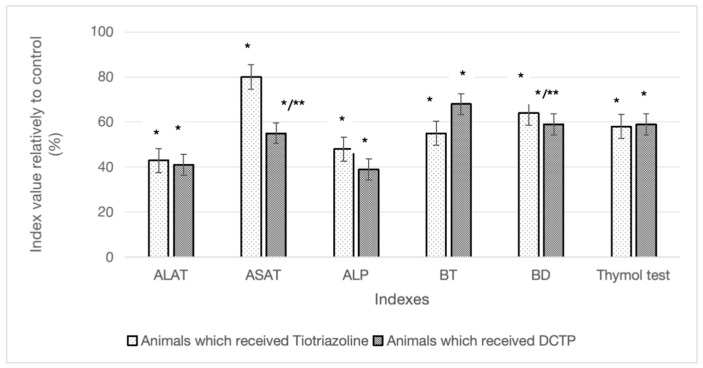
Differences in the studied biochemical parameters in experimental animals that received TTZ (Thiotriazoline) and DCTP in percentages compared to the control. * Significant difference from group I (control) (*p* < 0.05). ** Significant difference from group II (CCl_4_) (*p* < 0.05).

The AST activity in rats with experimental hepatitis after the administration of TTZ (group III) and DCTP (group IV) significantly decreased by 20.4% and 43.0%, respectively, compared to group II (CCl_4_), which may indicate the regeneration of liver mitochondria (Table 1). The administration of DCTP to animals (group IV) led to positive changes in AST compared to group III; the difference between the indicators was 28.4% (Figure 2). It should be noted that the obtained results on the effect of the substance on ALT and AST levels are consistent with the findings of the preliminary study [15]. 

The value of the De Ritis coefficient after tetrachloromethane administration to the experimental animals increased significantly in group III by 91.8% and in group IV by 40.9% compared to group II (CCl_4_) (Table 1). It should be noted that the effect of TTZ and DCTP on animals with hepatitis was different according to this indicator. In the experimental group III, it increased by 26.5% (*p* ≤ 0.05) compared to group IV (Table 1).

As is well known, an increase in the activity of alkaline phosphatase in the blood serum is used as a marker of cholestasis. The activity of this enzyme in animals with hepatitis treated with TTZ (group III) and DCTP (group IV) decreased compared to animals with hepatitis (group II (CCl_4_)) by 55.5% and 60.8%, respectively, and did not differ from the control (group I) (Table 1).

It was also shown that the level of both total and direct bilirubin increased in the blood of animals in group II (CCl_4_). The total bilirubin content in the groups of animals that received TTZ (group III) and DCTP (group IV) significantly decreased relative to the control by 46.0% and 32.7%, respectively. It was found that after the administration of TTZ (group III) and DCTP (group IV) to animals, a decrease in the direct bilirubin content in the serum was observed, by 40.9% and 36.4%, respectively (Figure 2). In the groups of animals with hepatitis that received TTZ (group III) and DCTP (group IV), there was a decrease in the thymol test index by 58.3% and 58.8%, respectively, compared to group II (CCl_4_).

### 3.2. Some Indicators of the Prooxidant–Antioxidant System in the Studied Groups of Animals

The results of the study showed that against the background of the action of CCl_4_, the content of diene conjugates in the liver increased by 2 times compared to group I (control) animals, and Schiff bases by 3 times (Table 2).

**Figure 3:**
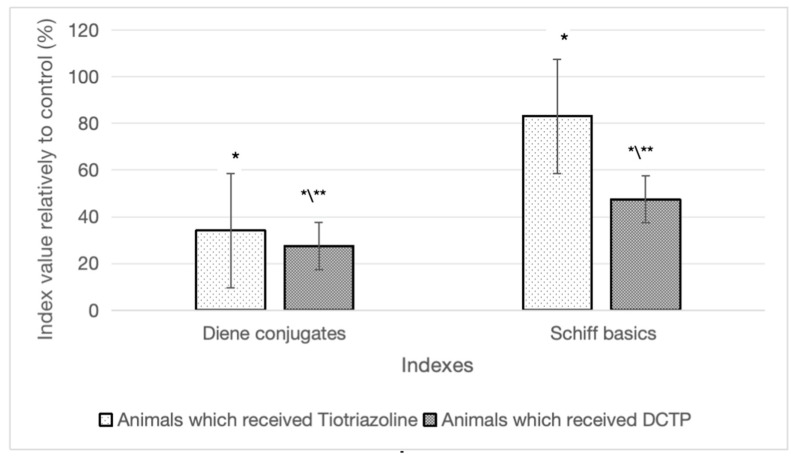
Differences in the content of diene conjugates and Schiff bases in animals with hepatitis that received TTZ (Thiotriazoline) and DCTP, as a percentage compared to group II (CCl_4_). * Significant difference from group I (control) (*p* < 0.05). ** Significant difference from group II (CCl_4_) (*p* < 0.05).

In the case where animals with hepatitis received DCTP (group IV) at a dose of 6 mg/100 g and TTZ (group III) at a dose of 10 mg/100 g, there was a decrease in the amount of diene conjugates in the liver by 43.2% and 29.2%, respectively, compared to group II (CCl_4_). The content of Schiff bases in the liver in these groups of animals decreased by 59% and 28.4%, respectively, compared to group II (CCl_4_) (Figure 3).

It should be noted that the effects of the studied compounds (TTZ and DCTP) did not lead to the restoration of the Schiff base content to the level of group I (control) animals (Table 2). These indicators remained higher by 62.5% and 22.6%, respectively, compared to the control (group I). Consequently, the action of DCTP provided a more pronounced effect on this indicator compared to Thiotriazoline.

**Figure 4:**
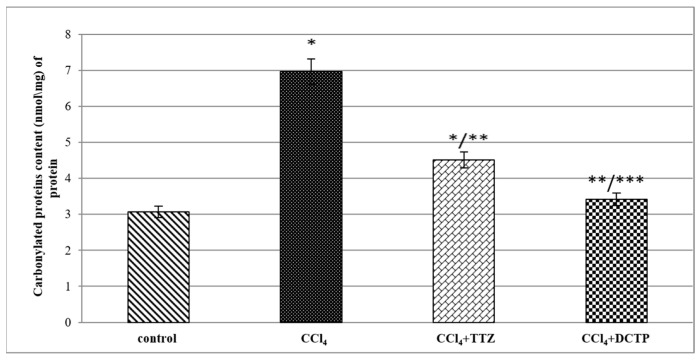
Carbonyl protein content in the liver homogenate of control rats (group I), rats with hepatitis (group II), and rats with hepatitis that received Thiotriazoline (group III) and DCTP (group IV). Mean values and standard errors are shown. There were 10 surviving rats in the control group, 7 surviving rats in group II (CCl_4_), 8 surviving rats in group III (CCl_4_ + TTZ), and 10 surviving rats in group IV (CCl_4_ + DCTP). * Significant difference from group I (control) (*p* < 0.05). ** Significant difference from group II (CCl_4_) (*p* < 0.05). *** Significant difference between group III (CCl_4_ + TTZ) and group IV (CCl_4_ + DCTP) (*p* < 0.05).

The results of the conducted studies on the content of carbonylated proteins showed that their amount in the liver with hepatitis (group II) was increased compared to the control (group I) by 2.3 times (Figure 4). This indicates an increase in the rate of the free radical oxidation of proteins and the disruption of the antioxidant system. In the case where TTZ was administered to animals with hepatitis (group III), this was accompanied by a decrease in carbonylated proteins in the liver by 35%, and if they received DCTP (group IV), the amount of oxidized proteins decreased by 50.8% (Figure 4). Consequently, DCTP had a more pronounced effect in reducing the concentration of carbonylated proteins in the liver with hepatitis compared to TTZ (group III), and this was 24.3% (Figure 4).

**Figure 5:**
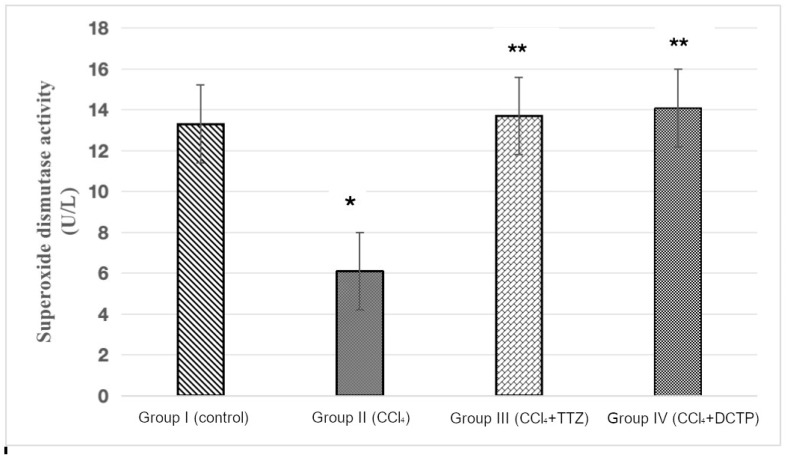
Superoxide dismutase activity in liver homogenate of control rats (group I), rats with hepatitis (group II), and rats with hepatitis that received Thiotriazoline (group III) and DCTP (group IV). Mean values and standard errors are shown. There were 10 surviving rats in the control group, 7 surviving rats in group II (CCl_4_), 8 surviving rats in group III (CCl_4_ + TTZ), and 10 surviving rats in group IV (CCl_4_ + DCTP). * Significant difference from group I (control) (*p* < 0.05). ** Significant difference from group II (CCl_4_) (*p* < 0.05).

These results correlate with the increase in the activity of antioxidant enzymes in animals receiving the studied compounds. It was found that the SOD activity in animals with hepatitis (group II) was lower than the control (group I) by 53.3% (Figure 5). The administration of TTZ (group III) and DCTP (group IV) to animals with experimental hepatitis was accompanied by the restoration of SOD activity to the level of the control rats (Figure 5).

### 3.3. Immunohistological and Cytological Indices in the Studied Groups of Animals

**Figure 6:**
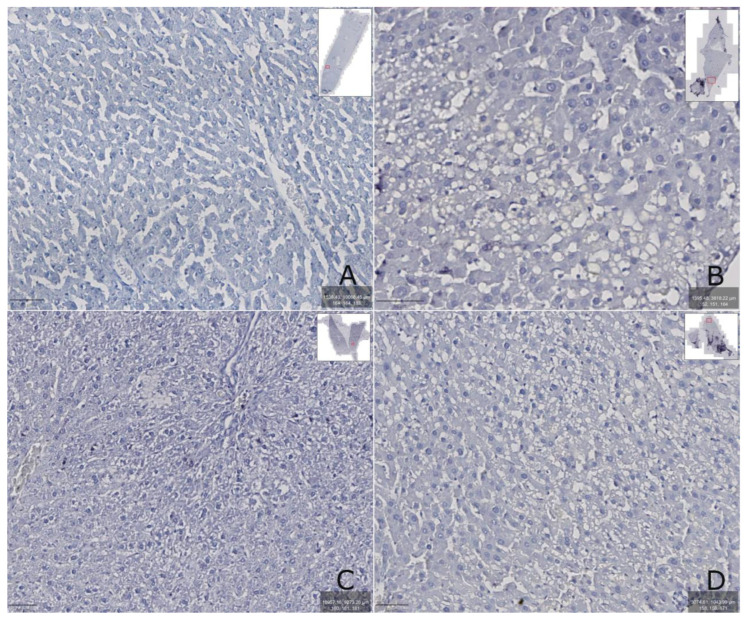
The immunohistochemical determination of Nrf2 protein in rat liver: (**A**)—group I (control); (**B**)—group II (CCl_4_); (**C**)—group III (CCl_4_ + TTZ); (**D**)—group IV (CCl_4_ + DCTP). The prepared preparations were scanned with a ZEISS Axioscan 7 scanner, and the images were enlarged at 400–600×.

The immunohistochemical determination of Nrf2 protein in rats with hepatitis (group II) showed that its amount was increased compared to group I (Figure 6B), which may indicate the manifestation of oxidative stress in the liver tissue. When TTZ was administered (group III), the Nrf2 protein remained elevated compared to the control group of animals (Figure 6C). At the same time, in animals receiving DCTP (group IV), the content of Nrf2 protein in the liver was reduced compared to group III receiving TTZ and did not differ from the control (Figure 6D). It should be noted that rats receiving an aqueous solution of DCTP to correct toxic damage did not have a completely restored structure of the liver lobule; however, compared to the control group and the group of rats receiving a TTZ solution to correct toxic changes, regeneration of the structure of hepatocytes and tissues was determined (Figure 6D).

**Figure 7:**
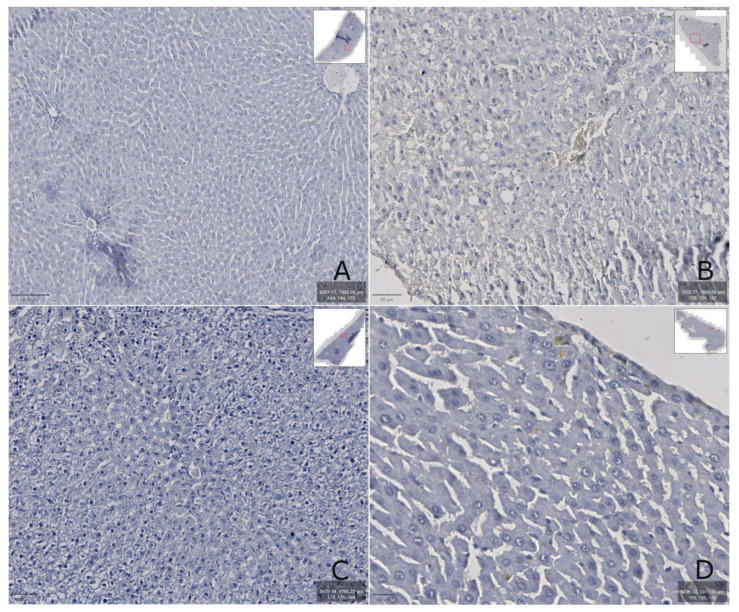
The immunohistochemical determination of LCN2 content in rat liver: (**A**)—group I (control); (**B**)—group II (CCl_4_); (**C**)—group III (CCl_4_ + TTZ); (**D**)—group IV (CCl_4_ + DCTP). The prepared preparations were scanned with a ZEISS Axioscan 7 scanner, and the images were enlarged at 400–600×.

It was found that the LCN2 content in rats increased after the CCl_4_ injection (group II) (Figure 7B) and correlated with increased AST and ALT activity in the blood serum (Table 1). In addition, toxic damage caused the appearance of severe degenerative changes (macrovesicular hepatocytes) and/or necrosis, as well as fatty degeneration (Figure 7B) accompanied by minor reversible changes (vacuolar hepatocytes) or severe degenerative changes (micro- and macrovesicular).

The toxic effects of CCl_4_ were manifested in massive central, perivenular, and intermediate hemorrhagic coagulative necrosis of the liver lobules compared to group I (control) animals (Figure 7B). They developed large quantities of fat droplets, which also indicates the development of acute toxic liver damage.

The administration of DCTP and the comparison drug, Thiotriazoline, had a significant hepatoprotective effect on acute liver injury, as evidenced by the analysis of biochemical and histological studies (Figure 7C,D).

**Figure 8:**
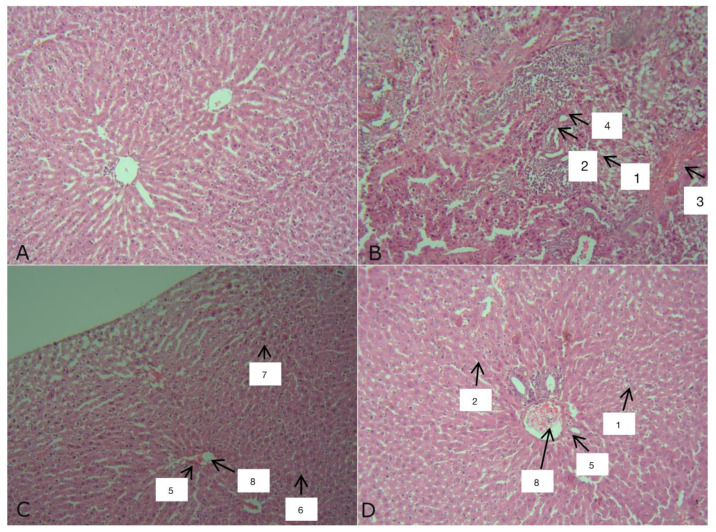
Rat liver: (**A**)—group I (control); (**B**)—group II (CCl_4_); (**C**)—group III (CCl_4_ + TTZ); (**D**)—group IV (CCl_4_ + DCTP). Hematoxylin–eosin staining. X200.

Histological examination of the livers of the rats in the control group showed the presence of the partial, weakly expressed autolysis of hepatocytes, while the structural organization of the liver lobules was maintained and blood filling was normal (Figure 8A). Such variations in structural changes in the liver reflect its reactions to the conditions in which the animals were kept and many factors unaccounted for that always occur.

In the initial stages of the development of toxic hepatitis induced by CCl_4_ (group II), an increase in the thickness of Glisson’s capsule, into which immunocompetent cells have been incorporated, is observed (Figure 8B). In the liver of rats, the partial autolysis of hepatocytes took place. (1) Discomplexation of the liver beams was often encountered. (2) The vessels were filled with blood (3), but the Disse space was reduced (4), in which Ito cells were often found. There were few fibroblasts, and endothelial cells were found in moderate quantities, most often near blood vessels. These minor structural changes in the liver, compared with the control, were accompanied by minor changes in the activity of specific liver enzymes. In such animals, there was no blood filling in the veins and arteries (3), and in some cases, hemorrhage in the central vein was observed. The integrity of the endothelium was impaired. The liver lobules were damaged.

In the group III (CCl_4_ + TTZ) solution (Figure 8C), the vessels were filled with blood (8). The Disse space was enlarged (3), and Ito cells were quite common (4). Endothelial cells (5), small amounts of lymphocytes (6), and fibroblasts (7) were visible around blood vessels (Figure 8D).

In animals with toxic hepatitis treated with DCTP (group IV) (Figure 8D), pronounced discomplexation of the liver trabeculum (1) and complete autolysis of hepatocytes (2) were observed. The nuclei were approximately the same shape and size, i.e., round. Around the central vein and blood artery (8), a moderate number of lymphocytes could be observed (6). The Disse space was reduced (3), and Ito cells (4) were seen most often in the area of the liver lobules. Numerous fibroblasts and endotheliocytes (5) were found in the preparation, most often located near the vessels. Multiple ruptures were observed throughout the preparation (Figure 8D).

### 3.4. Some Physiological Indicators in the Studied Groups of Animals

The conducted studies showed that when the hepatotoxic agent CCl_4_ was introduced, the lethality of animals increased depending on the chemical damage to liver cells (hepatocytes). The survival rates of the animals were as follows: control group (group I)—10 rats; CCl_4_ (group II)—7 rats; CCl_4_ + TTZ (group III)—8 rats; and CCl_4_ + DCTP (group IV)—10 rats. The highest survival rate (100%) was noted after the administration of the DCTP compound (group IV).

**Figure 9:**
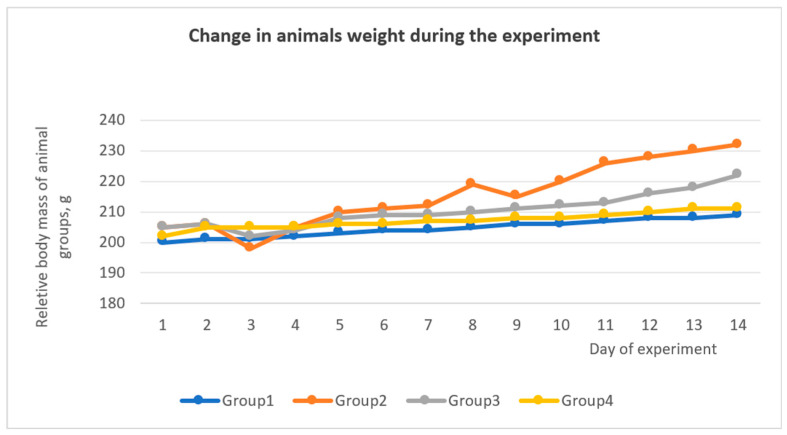
Change in rat weight during the experiment.

**Figure 10:**
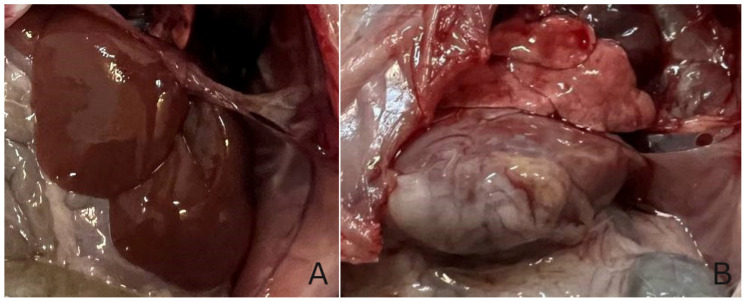
Photo of the liver in normal conditions and with pathology: (**A**)—group I (control); (**B**)—group II (CCl_4_).

Also, during the experiment, a change in the weight of the experimental animals was observed (Figure 9); in particular, an increase in the average weight was observed in group II (CCl_4_). A change in weight was observed in all groups in the first days after the administration of CCl_4_ (Figure 2), and after 5–6 days, the weight began to recover. Weight growth in the group II (CCl_4_) was observed due to the formation of adhesions between liver particles, the formation of a capsule around the liver (Figure 10B), and a general increase in liver weight by 30%.

The liver of rats from group III (CCl_4_ + TTZ) and group IV (CCl_4_ + DCTP) were anatomically no different from group I (control).

## 4. Discussion

The liver plays an important role in detoxification and metabolic processes, but despite its strong regenerative capacity, it is also responsible for damage caused by chemicals, drugs, and environmental toxicants [31]. It is known that CCl_4_ is often used to model acute hepatitis in experimental animals due to the induction of oxidative stress, lipid peroxidation, the formation of carbonylated proteins, and the activation of inflammatory processes [32].

During oxidative stress, toxic metabolites of CCl_4_ cause a decrease in enzyme activity and a disruption of antioxidant protection (SOD, ALAT, ASAT, ALP, BT, BD, thymol test) while simultaneously enhancing the manifestation of prooxidant markers (carbonyl proteins, Schiff bases, diene conjugates, O2-). This leads to the development of oxidative stress and damage to liver cells [15,33].

This study revealed a high content of carbonylated proteins in the liver mitochondria of animals under oxidative stress, which led to a loss in their biological activity. In the scientific literature, much attention has been paid to the study of prooxidant changes, where the significant role of the oxidative modification of proteins (OMP) in the development of liver disorders is emphasized. OMP is considered one of the first and most reliable markers of tissue damage in pathologies associated with free radicals [34]. Carbonylated proteins are relatively stable compounds that are formed because of the metal-catalyzed oxidation of proline, arginine, lysine or threonine residues, leading to the formation of Michael adducts [35].

There are many pathways for the formation of carbonylated proteins in a cell. They can be formed not only through direct oxidation but also through the participation of lipid peroxidation products, as well as in the glycation or glycoxidation of lysine amino groups [36]. An increased level of carbonylated proteins (CPs) is traditionally associated with carbonyl stress [37], which is a significant pathogenetic factor. This process leads to the suppression of enzymatic activity, changes in the structural organization of membrane proteins, and the disruption of protein folding, which is especially noticeable in the carbonylation of Hsp90 chaperones [38,39].

However, studies have shown that in addition to pathological effects, carbonylated proteins also perform important physiological functions. They participate in the regulation of gene expression associated with antioxidant protection (via the NF-E2-related factor 2) [40] and also play a role in signaling mechanisms regulating various cellular processes [41].

During the lipid peroxidation phase, unneutralized toxic CCl_4_·radicals form covalent bonds with the proteins and lipids of hepatocyte membranes, as well as with the membranes of mitochondria and the endoplasmic reticulum. The reactive CCl_3_O_2_ radical then initiates the lipid peroxidation process, which leads to morphological and functional damage to liver cells [42].

In the inflammatory phase, CCl_4_ free radicals cause hypertrophy and hyperplasia of Kupffer cells, which in turn begin to produce and secrete many toxic and proinflammatory compounds. This increases damage to liver parenchymal cells [43]. During oxidative stress, toxic CCl_4_ metabolites contribute to an increase in the activity of liver damage markers (Nrf2 and Lipocalin-2) by triggering lipid peroxidation processes and the destruction of polyunsaturated fatty acids and phospholipids [33].

Lipocalin-2 (LCN2) acts as an early biomarker of liver inflammation, the level of which correlates with the severity of organ damage. Acute inflammatory and toxic liver damage, as well as secreted proinflammatory cytokines (IL-1β, IL-6, TNF-α), synthesized by activated Kupffer cells, are powerful stimulators of LCN2 expression in damaged hepatocytes. The highest immunohistochemical detection of LCN2 is observed in the centrilobular zone, which coincides with the morphological distribution of damaged hepatocytes in the liver acinus.

At the same time, released LCN2 stimulates Kupffer cells to release other chemokines, which attract neutrophils and monocytes to the area of toxic damage and inflammation [44,45].

The presented results demonstrate that the acute toxic effects of CCl_4_ caused a significant increase in the activity of enzymes (ALT, AST, alkaline phosphatase, SOD), prooxidant markers of LPO (diene conjugates and Schiff bases), and carbonylated proteins, as well as the levels of direct and total bilirubin. The level of gamma-glutamyltransferase (GGT) was not assessed in the present study due to the use of an acute model of hepatitis. These indicators significantly exceeded the values of the control group without treatment, which is consistent with the data from other studies [46,47,48].

The resulting shifts in liver metabolism were normalized using the reference drug Thiotriazoline (a hepatoprotective agent from the triazole group with antioxidant action) [49]. The administration of DCTP to rats with experimental acute hepatitis led to a decrease in the cytolytic process and the regeneration of the functional activity of the liver, and, in terms of these indicators, it was not inferior to the well-known drug TTZ, and in terms of other indicators (the level of ASAT, diene conjugates, Schiff bases, and carbonylated proteins), it even exceeded TTZ.

The occurrence and development of toxic effects in the liver are caused by the activation of lipid peroxidation processes, the oxidative modification of proteins and nucleic acids, and oxidative damage to cell membranes, which leads to liver dysfunction [33], and this is the main factor in the formation of oxidative stress [50].

In the next stage of this work, the influence of CCl_4_ on some indices of the prooxidant–antioxidant system in the liver [15] in the studied groups of animals was determined. For this purpose, the concentrations of primary (diene conjugates) and final (Schiff bases) substances were determined.

Under the influence of oxidative stress, the administration of the DCTP compound leads to a significant decrease in biochemical parameters, has powerful antioxidant activity, blocks oxidative stress, prevents cell death by preventing OMP, and can activate antioxidant enzyme systems by activating SOD and inhibiting active oxygen species (AOS).

Studies conducted on an experimental model of CCl_4_-induced hepatitis have proven the ability of this synthesized substance to reduce the concentration of LPO products. Data have been obtained on the ability of the DCTP substance to stimulate the protein-synthesizing function of the liver, prevent protein catabolism, and inhibit inflammatory reactions and the cytolysis of hepatocytes, because of which the bile secretory and detoxifying functions of the liver are restored.

Consequently, the studied drugs reduced the manifestation of oxidative stress in animals with hepatitis, and this was expressed to a greater extent for DCTP compared to Thiotriazoline.

The restoration of SOD activity may be one of the reasons for the decrease in antioxidant activity. In the third and fourth groups of rats with experimental hepatitis, an increase in the SOD level was noted, indicating an increase in the compensatory mechanisms of antioxidant systems by 2.2 and 2.3 times compared to the control. With the administration of TTZ (group III) and the DCTP compound (group IV), the SOD indicator was restored to 13.56 ± 1.01 and 14.19 ± 0.96, respectively, which is equal to the value in the control rats (group I). (Figure 5).

Oxidative stress underlies most liver diseases, including pharmacological liver injury, viral hepatitis, and alcoholic hepatitis. The Kerch system, ECH-associated protein 1-NFE2-associated factor 2 (Keap1-C), is an important protective mechanism of cells and organisms against oxidative stress. Its regulation reduces drug-induced liver injury in rats. In addition, many natural Nrf2 activators regulate lipid metabolism and oxidative stress in hepatocytes, thereby reducing fatty liver disease in mice [51]. Increased Nrf2 is observed in acute hepatitis, fatty liver disease, and viral hepatitis and apoptosis [52]. Nrf2 also plays an important role in the activation of antioxidant enzymes, regulating their transcription [51].

Lipocalin-2 (LCN2) is expressed in pathological conditions such as intoxication, infection, inflammation, and other forms of cellular stress. Experimental liver injury causes the rapid and sustained formation of LCN2 by damaged hepatocytes. However, the exact biological function of LCN2 in the liver is still unknown. In this work, the content of LCN2 was determined through histological preparations in the studied liver samples [53].

The administration of the test substance significantly reduced the activity of specific damage biomarkers (Nrf2 and Lipocalin-2), which indicates its ability to protect hepatocyte membranes and organelles from the toxic effects of CCl_4_.

According to the literature, fibrotic changes involve a larger number of cells (fibroblasts, vascular cells, infiltrating immune cells, and biliary epithelial cells) [54]. Glisson’s capsule is a layer of interstitial tissue that can also be classified as visceral fascia [55,56,57]. It surrounds the liver and is continuous with the interstitial spaces and matrix surrounding the portal triads, likely playing a role in fluid exchange and cell migration in the hepatobiliary system [57,58,59]. The capsule is known to be constructed in such a way that it allows for diurnal variations in size due to changes in hepatocyte size after meals, as well as circadian regulation by 34% in mice and 10–15% in humans [60]. To allow for this size variability, the normal liver capsule is thin and well innervated and contains many elastic fibers, as is characteristic of the visceral fascia [61]. These characteristics create a unique niche in which different populations of both macrophages and fibroblasts coexist under normal physiological conditions [62]. As described, the visualization of the surface of the fibrous rat liver reveals altered collagen organization with a denser collagen network and a loss in the characteristic fiber waviness. These changes are highly dependent on disease severity [63].

Therefore, to predict the degree of liver fibrosis in toxic hepatitis, it is possible to analyze the presence of changes, namely the thickening of Glisson’s capsule, as a marker of fibrosis, which indicates changes in the capsular matrix.

Other rat studies have shown that in addition to changes in the matrix, the capsular cell population also changed, with mesothelial cells and fibroblasts migrating into the liver parenchyma and contributing to the formation of a myofibroblast population [62,64,65]. The capsule is an active site of pathology in rat liver fibrosis, with mesothelial cells, fibroblasts, and macrophages embedded in a thin layer of the matrix.

Our study showed that animals treated with DCTP at the initial stages of fibrosis development had minor histological changes, indicating its pronounced hepatoprotective effect. The most pronounced deviations in this group were the thickening of Glisson’s capsule.

The dynamics of the relative liver mass on the third to fifth day after the administration of tetrachloromethane in all groups of animals were also observed. Changes were observed in the control group of animals, which were not subject to a correction of pathological conditions. In the groups of animals subjected to treatment, the mass indices were at the control level, coinciding with the studies of other authors [66].

The hepatoprotective effect of the studied compound is likely due to its structural similarity to tetrahydrofolic acid, an essential component of redox processes and a participant in the biosynthesis of nitrogenous bases of nucleotides and amino acids [67,68,69].

In clinical practice, other hepatoprotective agents such as silymarin, ursodeoxycholic acid, N-acetylcysteine, and metformin are widely used [70,71,72]. Metformin, a well-known antidiabetic drug, has demonstrated potential in reducing liver fibrosis through its effects on metabolic pathways, inflammation, and the activation of AMPK [73,74,75,76].

Recent studies also suggest that genetic factors, including polymorphisms in genes involved in fibrogenesis and drug metabolism, may influence individual responses to hepatoprotective therapy [77,78,79,80]. The effectiveness of these agents may vary depending on the etiology of liver injury and the stage of disease progression [81,82,83]. It is important to consider that infectious diseases, particularly viral hepatitis B and C, as well as COVID-19 [84,85,86,87], can significantly accelerate the development of fibrotic changes and alter the therapeutic response to hepatoprotective agents [88,89,90].

In addition, comorbid conditions such as obesity, diabetes mellitus [91,92], and cardiovascular diseases [93,94] may exacerbate liver injury through metabolic and inflammatory mechanisms [95,96,97]. These comorbidities can also reduce the effectiveness of therapeutic agents and require an individualized treatment approach [98,99,100].

Therefore, future studies should evaluate the impact of concomitant pathologies and infectious agents, particularly SARS-CoV-2, on the progression of fibrosis and the efficacy of hepatoprotective therapies.

## 5. Conclusions

The liver damage induced by CCl_4_ in the experimental animals resulted in cell membrane damage, increased LPO, and increased AST, ALT, ALP, total and direct bilirubin, and thymol test values. The resulting shifts in liver metabolism were normalized by the administration of the studied pteridine derivative, which exceeded the effect of the TTZ reference solution in terms of its effect on the levels of AST, DC, Schiff bases, and carbonylated proteins.

It was shown that the studied substance, DCTP, significantly reduces acute hepatotoxic effects caused by CCl_4_, as evidenced by the decrease in the level of lipid peroxidation and prooxidant markers, the normalization of liver biochemical markers, the regeneration of the liver architecture, the limitation of inflammatory effects, the decrease in Nrf2 and Lipocalin-2 markers, and the induction of liver antioxidant enzymes.

Our results show the antioxidant and hepatoprotective properties of the studied S-substituted pteridine. Detailed data on the studied substance and the mechanisms underlying them require further research.

## Figures and Tables

**Table 1:** Biochemical parameters of blood serum of animals in the studied groups.

Indicators	Experimental Groups			
	Group I (Control)	Group II (CCl_4_)	Group III (CCl_4_ + TTZ)	Group IV (CCl_4_ + DCTP)
ALT (Units/L)	42.60 ± 2.80	147.90 ± 9.49 *	63.03 ± 10.50 */**	60.50 ± 6.80 */**
AST (Units/L)	78.10 ± 5.30	162.70 ± 12.80 *	129.50 ± 11.49 */**	92.70 ± 9.80 */**/***
De Ritis coefficient	1.80 ± 0.10 *	1.10 ± 0.03 *	2.12 ± 0.49 **	1.56 ± 0.29 */**/***
Alkaline phosphatase (Units/L)	90.50 ± 15.80	228.30 ± 44.70 *	101.50 ± 5.45 **	89.40 ± 21.00 **
Total bilirubin (mmol/L)	9.60 ± 1.80	19.18 ± 1.80 *	10.35 ± 1.05 **	12.90 ± 2.10 **
Direct bilirubin (mmol/L)	3.30 ± 0.10	5.58 ± 0.78 *	3.56 ± 0.19 **	3.30 ± 0.40 **
Thymol test (Sh)	0.90 ± 0.05	4.62 ± 0.56 *	1.93 ± 0.46 **	1.90 ± 0.20 **

Mean values and standard errors are presented. There were 10 surviving rats in the control group (group I), 7 surviving rats in group II (CCl_4_), 8 surviving rats in group III (CCl_4_ + TTZ) at a dose of 10 mg/100 g, and 10 surviving rats in group IV (CCl_4_ + DCTP). * Significant difference from group I (control) (*p* < 0.05). ** Significant difference from group II (CCl_4_) (*p* < 0.05). *** Significant difference from group III (CCl_4_ + TTZ) (*p* < 0.05).

**Table 2:** Content of diene conjugates and Schiff bases in the liver in the studied groups of animals: control animals (group I); animals with hepatitis (group II); animals with hepatitis that received Thiotriazoline (group III); and animals with hepatitis that received DCTP (group IV).

Animal Group	Diene Conjugates (nmol/g)	Schiff Bases (nmol/g)
Group I (control)	25.19 ± 2.38	38.80 ± 17.69
Group II (CCl_4_)	48.34 ± 1.06 *	116.00 ± 28.33 *
Group III (CCl_4_ + TTZ)	34.22 ± 2.63 **	83.11 ± 14.94 **
Group IV (CCl_4_ + DCTP)	27.48 ± 1.34 **/***	47.56 ± 10.67 **/***

The mean values and standard errors are presented. There were 10 surviving rats in the control group, 7 surviving rats in group II (CCl4), 8 surviving rats in group III (CCl4 + TTZ), and 10 surviving rats in group IV (CCl_4_ + DCTP). * Significant difference from group I (control) (*p* < 0.05). ** Significant difference from group II (CCl_4_) (*p* < 0.05). *** Significant difference between group III (CCl_4_ + TTZ) and group IV (CCl_4_ + DCTP) (*p* < 0.05).

## Data Availability

Data is contained within the article.

## References

[1] Asrani S.K., Devarbhavi H., Eaton J., Kamath P.S. (2019). Burden of Liver Diseases in the World. J. Hepatol..

[2] Conway R., Carey J.J. (2017). Risk of Liver Disease in Methotrexate Treated Patients. World J. Hepatol..

[3] O’Shea R.S., Dasarathy S., McCullough A.J. (2009). Alcoholic Liver Disease. Hepatology.

[4] Saeter G., Lee C.-Z., Schwarze P.K., Ous S., Chen D.-S., Sung J.-L., Seglen P.O. (1988). Changes in Ploidy Distributions in Human Liver Carcinogenesis. J. Natl. Cancer Inst..

[5] Reyes-Gordillo K., Shah R., Muriel P. (2017). Oxidative Stress and Inflammation in Hepatic Diseases: Current and Future Therapy. Oxid. Med. Cell. Longev..

[6] Nolan J.P. (2010). The Role of Intestinal Endotoxin in Liver Injury: A Long and Evolving History. Hepatology.

[7] Hoffman D.R., Marion D.W., Cornatzer W.E., Duerre J.A. (1980). S-Adenosylmethionine and S-Adenosylhomocysteine Metabolism in Isolated Rat Liver. J. Biol. Chem..

[8] Lan X., Field M.S., Stover P.J. (2018). Cell Cycle Regulation of Folate-Mediated One-Carbon Metabolism. Wiley Interdiscip. Rev. Syst. Biol. Med..

[9] Hine C., Harputlugil E., Zhang Y., Ruckenstuhl C., Lee B.C., Brace L., Longchamp A., Treviño-Villarreal J.H., Mejia P., Ozaki C.K. (2015). Endogenous Hydrogen Sulfide Production is Essential for Dietary Restriction Benefits. Cell.

[10] Sanderson S.M., Gao X., Dai Z., Locasale J.W. (2019). Methionine Metabolism in Health and Cancer: A Nexus of Diet and Precision Medicine. Nat. Rev. Cancer.

[11] DrugBank DB12687. https://go.drugbank.com/drugs/DB12687.

[12] Kazunin M.S., Groma N.V., Nosulenko I.S., Kinichenko A.O., Antypenko O.M., Shvets V.M., Voskoboinik O.Y., Kovalenko S.I. (2022). Thio-Containing Pteridines: Synthesis, Modification, and Biological Activity. Arch. Pharm..

[13] Lohvinenko N., Shvets V., Berest G., Nosulenko I., Voskoboinik O., Severina H., Okovytyy S., Kovalenko S. (2024). Prospects for the Use of Sulfur-Containing Pteridines in Toxic Liver Damage. Regul. Mech. Biosyst..

[14] Milstien S., Kapatos G., Levine R.A., Shane B. (2002). Chemistry and Biology of Pteridines and Folates.

[15] Groma N., Berest G., Antypenko O., Voskoboinik O., Kopiika V., Kovalenko S., Shvets V. (2023). Evaluation of the Toxicity and Hepatoprotective Properties of New S-Substituted Pteridins. Curr. Issues Pharm. Med. Sci..

[16] Kozhem’yakin Y.M., Khromov O.S., Filonenko M.A. (2002). Scientific and Practical Recommendations for the Development of Laboratory Creatures and Robots.

[17] (1986). European Convention for the Protection of Vertebrate Animals Used for Experimental and Other Scientific Purposes.

[18] Stefanov O.V. (2001). Preclinical Study of Drugs (Methodical Recommendation).

[19] Bhakuni G.S., Bedi O., Bariwal J., Deshmukh R., Kumar P. (2015). Animal Models of Hepatotoxicity. Inflamm. Res..

[20] Mazur I.A., Voloshin N.A., Chekman I.S. (2005). Thiotriazolin: Pharmacological Aspects and Clinical Application.

[21] Recknagel R.O., Ghoshal A.K. (1966). Quantitative Estimation of Peroxidative Degeneration of Rat Liver Microsomal and Mitochondrial Lipids After Carbon Tetrachloride Poisoning. Exp. Mol. Pathol..

[22] Symons M.C.R., Diplock A.T., Rice-Evans C.A. (1991). Techniques in Free Radical Research.

[23] Kostyuk V.A. (1990). A Simple and Sensitive Method for Determining Superoxide Dismutase Activity Based on the Oxidation Reaction of Quercetin. Questions Med. Chem..

[24] Levine R.L., Stadtman E.R. (2001). Oxidative Modification of Proteins During Aging. Exp. Gerontol..

[25] Vlizlo V.V., Fedoruk R.S., Ratych I.B., Vishurt O.I., Sharan M.M., Vudmaska I.V., Fedorovych E.I. (2012). Laboratory Research Methods in Biology, Animal Husbandry and Veterinary Medicine: A Guide.

[26] Schumann G., Klauke R., Canalias F., Bossert-Reuther S., Franck P.F., Gella F.-J., Jørgensen P.J., Kang D., Lessinger J.-M., Panteghini M. (2011). IFCC Primary Reference Procedures for the Measurement of Catalytic Activity Concentrations of Enzymes at 37 °C. Part 9: Reference Procedure for the Measurement of Catalytic Concentration of Alkaline Phosphatase. Clin. Chem. Lab. Med..

[27] Davydov V.V., Shvets V.N. (2011). Guide to Practical Classes in Biological Chemistry (for Students of Medical Schools III-IV Level of Accreditation).

[28] Jendrassik L., Grof P. (1938). Colorimetric Method of Determination of Bilirubin. Biochem. Z..

[29] Menshikov V.V. (1987). Laboratory Research Methods in Clinic. Meditsina.

[30] Borkham-Kamphorst E., Kovalenko E., Van Roeyen C.R., Gassler N., Bomble M., Ostendorf T., Floege J., Gressner A.M., Weiskirchen R. (2008). Platelet-Derived Growth Factor Isoform Expression in Carbon Tetrachloride-Induced Chronic Liver Injury. Lab. Investig..

[31] Zhang C.-Y., Yuan W.-G., He P., Lei J.-H., Wang C.-X. (2016). Liver Fibrosis and Hepatic Stellate Cells: Etiology, Pathological Hallmarks and Therapeutic Targets. World J. Gastroenterol..

[32] Ighodaro O.M., Asejeje A.O., Adeosun A.M., Ujomu T.S., Olisedeme C.J. (2020). Dose and Time Dependent Effects of Intraperitoneal Administration of Carbon Tetrachloride (CCl_4_) on Blood Lipid Profile in Wistar Rats. EC Pharmacol. Toxicol..

[33] Manhar N., Singh S.K., Yadav P., Bishnolia M., Khurana A., Bhatti J.S., Navik U. (2025). Methyl Donor Ameliorates CCl_4_-Induced Nephrotoxicity by Inhibiting Oxidative Stress, Inflammation, and Fibrosis Through the Attenuation of Kidney Injury Molecule 1 and Neutrophil Gelatinase-Associated Lipocalin Expression. J. Biochem. Mol. Toxicol..

[34] Stefan N., Kantartzis K., Häring H.-U. (2008). Causes and Metabolic Consequences of Fatty Liver. Endocr. Rev..

[35] Akagawa M. (2021). Protein Carbonylation: Molecular Mechanisms, Biological Implications, and Analytical Approaches. Free Radic. Res..

[36] Friguet B., Bulteau A.-L., Chondrogianni N., Conconi M., Petropoulos I.-B. (2006). Protein Degradation by the Proteasome and Its Implications in Aging. Ann. N. Y. Acad. Sci..

[37] Davydov V.V., Bozhkov A.I., Kulchitsky O.K. (2012). Physiological and Pathophysiological Role of Endogenous Aldehydes.

[38] Carbone D.L., Doorn J.A., Kiebler Z., Ickes B.R., Petersen D.R. (2005). Modification of Heat Shock Protein 90 by 4-Hydroxynonenal in a Rat Model of Chronic Alcoholic Liver Disease. J. Pharmacol. Exp. Ther..

[39] Carbone D.L., Doorn J.A., Kiebler Z., Petersen D.R. (2005). Cysteine Modification by Lipid Peroxidation Products Inhibits Protein Disulfide Isomerase. Chem. Res. Toxicol..

[40] Walters D.M., Cho H.-Y., Kleeberger S.R. (2008). Oxidative Stress and Antioxidants in the Pathogenesis of Pulmonary Fibrosis: A Potential Role for Nrf2. Antioxid. Redox Signal..

[41] Wong C.M., Cheema A.K., Zhang L., Suzuki Y.J. (2008). Protein Carbonylation as a Novel Mechanism in Redox Signaling. Circ. Res..

[42] Knockaert L., Berson A., Ribault C., Prost P.-E., Fautrel A., Pajaud J., Lepage S., Lucas-Clerc C., Bégué J.-M., Fromenty B. (2011). Carbon Tetrachloride-Mediated Lipid Peroxidation Induces Early Mitochondrial Alterations in Mouse Liver. Lab. Investig..

[43] (2023). VI International Scientific and Theoretical Conference «Sectoral Research XXI: Characteristics and Features».

[44] Borkham-Kamphorst E., Drews F., Weiskirchen R. (2011). Induction of Lipocalin-2 Expression in Acute and Chronic Experimental Liver Injury Moderated by Pro-Inflammatory Cytokines Interleukin-1β Through Nuclear Factor-κB Activation. Liver Int..

[45] Dahl S.L., Woodworth J.S., Lerche C.J., Cramer E.P., Nielsen P.R., Moser C., Thomsen A.R., Borregaard N., Cowland J.B. (2018). Lipocalin-2 Functions as Inhibitor of Innate Resistance to Mycobacterium tuberculosis. Front. Immunol..

[46] DeCicco L.A., Rikans L.E., Tutor C.G., Hornbrook K.R. (1998). Serum and Liver Concentrations of Tumor Necrosis Factor α and Interleukin-1β Following Administration of Carbon Tetrachloride to Male Rats. Toxicol. Lett..

[47] Teschke R., Vierke W., Goldermann L. (1983). Carbon Tetrachloride (CCl_4_) Levels and Serum Activities of Liver Enzymes Following Acute CCl4 Intoxication. Toxicol. Lett..

[48] Teschke R. (2018). Liver Injury by Carbon Tetrachloride Intoxication in 16 Patients Treated with Forced Ventilation to Accelerate Toxin Removal via the Lungs: A Clinical Report. Toxics.

[49] Stepanov Y.M., Kosynskaya S.I. (2010). The Use of Thiotriazoline in Patients with Chronic Liver Diseases. Zaporizhzhya Med. J..

[50] Bernabeu-Wittel M., Gómez-Díaz R., González-Molina Á., Vidal-Serrano S., Díez-Manglano J., Salgado F., Soto-Martín M., Ollero-Baturone M., Researchers O.B.O.T.P. (2020). Oxidative Stress, Telomere Shortening, and Apoptosis Associated to Sarcopenia and Frailty in Patients with Multimorbidity. J. Clin. Med..

[51] Zhou J., Zheng Q., Chen Z. (2022). The Nrf2 Pathway in Liver Diseases. Front. Cell Dev. Biol..

[52] Yamashita Y., Ueyama T., Nishi T., Yamamoto Y., Kawakoshi A., Sunami S., Iguchi M., Tamai H., Ueda K., Ito T. (2014). Nrf2-Inducing Anti-Oxidation Stress Response in the Rat Liver—New Beneficial Effect of Lansoprazole. PLoS ONE.

[53] Asimakopoulou A., Weiskirchen S., Weiskirchen R. (2016). Lipocalin 2 (LCN2) Expression in Hepatic Malfunction and Therapy. Front. Physiol..

[54] Llewellyn J., Fede C., Loneker A.E., Friday C.S., Hast M.W., Theise N.D., Furth E.E., Guido M., Stecco C., Wells R.G. (2023). Glisson’s Capsule Matrix Structure and Function Is Altered in Patients with Cirrhosis Irrespective of Etiology. JHEP Rep..

[55] Stecco C., Sfriso M.M., Porzionato A., Rambaldo A., Albertin G., Macchi V., De Caro R. (2017). Microscopic Anatomy of the Visceral Fasciae. J. Anat..

[56] Allen W.E. (2009). Terminologia Anatomica: International Anatomical Terminology and Terminologia Histologica: International Terms for Human Cytology and Histology. J. Anat..

[57] Benias P.C., Wells R.G., Sackey-Aboagye B., Klavan H., Reidy J., Buonocore D., Miranda M., Kornacki S., Wayne M., Carr-Locke D.L. (2018). Structure and Distribution of an Unrecognized Interstitium in Human Tissues. Sci. Rep..

[58] Hayashi S., Murakami G., Ohtsuka A., Itoh M., Nakano T., Fukuzawa Y. (2008). Connective Tissue Configuration in the Human Liver Hilar Region with Special Reference to the Liver Capsule and Vascular Sheath. J. Hepatobiliary-Pancreat. Surg..

[59] Wang J., Kubes P. (2016). A Reservoir of Mature Cavity Macrophages That Can Rapidly Invade Visceral Organs to Affect Tissue Repair. Cell.

[60] Wagner B.A., Venkataraman S., Buettner G.R. (2011). The Rate of Oxygen Utilization by Cells. Free Radic. Biol. Med..

[61] Acharya P., Chouhan K., Weiskirchen S., Weiskirchen R. (2021). Cellular Mechanisms of Liver Fibrosis. Front. Pharmacol..

[62] Guilliams M., Bonnardel J., Haest B., Vanderborght B., Wagner C., Remmerie A., Bujko A., Martens L., Thoné T., Browaeys R. (2022). Spatial Proteogenomics Reveals Distinct and Evolutionarily Conserved Hepatic Macrophage Niches. Cell.

[63] Xu S., Kang C.H., Gou X., Peng Q., Yan J., Zhuo S., Cheng C.L., He Y., Kang Y., Xia W. (2015). Quantification of Liver Fibrosis via Second Harmonic Imaging of the Glisson’s Capsule from Liver Surface. J. Biophotonics.

[64] Balog S., Li Y., Ogawa T., Miki T., Saito T., French S.W., Asahina K. (2019). Development of Capsular Fibrosis Beneath the Liver Surface in Humans and Mice. Hepatology.

[65] Li Y., Wang J., Asahina K. (2013). Mesothelial Cells Give Rise to Hepatic Stellate Cells and Myofibroblasts via Mesothelial-Mesenchymal Transition in Liver Injury. Proc. Natl. Acad. Sci. USA.

[66] Bozhkov A., Ionov I., Kurhuzova N., Novikova A., Katerynych O., Akzhyhitov R. (2022). Vitamin A Intake Forms Resistance to Hypervitaminosis A and Affects the Functional Activity of the Liver. Clin. Nutr. Open Sci..

[67] Salama S., Kue C.S., Mohamad H., Omer F., Ibrahim M.Y., Abdulla M., Ali H., Mariod A., Jayash S.N. (2022). Hepatoprotective Potential of a Novel Quinazoline Derivative in Thioacetamide-Induced Liver Toxicity. Front. Pharmacol..

[68] He Y., Xia Z., Yu D., Wang J., Jin L., Huang D., Ye X., Li X., Zhang B. (2019). Hepatoprotective Effects and Structure–Activity Relationship of Five Flavonoids against Lipopolysaccharide/D-Galactosamine-Induced Acute Liver Failure in Mice. Int. Immunopharmacol..

[69] Chandel N.S. (2021). Nucleotide Metabolism. Cold Spring Harb. Perspect. Biol..

[70] Halabitska I., Oksenych V., Kamyshnyi O. (2024). Exploring the Efficacy of Alpha-Lipoic Acid in Comorbid Osteoarthritis and Type 2 Diabetes Mellitus. Nutrients.

[71] Shan D., Dai S., Chen Q., Xie Y., Hu Y. (2023). Hepatoprotective Agents in the Management of Intrahepatic Cholestasis of Pregnancy: Current Knowledge and Prospects. Front. Pharmacol..

[72] Oliveira C., Cotrim H., Stefano J.T., Siqueira A., Salgado A., Parise E. (2019). N-Acetylcysteine and/or Ursodeoxycholic Acid Associated with Metformin in Non-Alcoholic Steatohepatitis: An Open-Label Multicenter Randomized Controlled Trial. Arq. Gastroenterol..

[73] Petakh P., Kamyshna I., Oksenych V., Kainov D., Kamyshnyi A. (2023). Metformin Therapy Changes Gut Microbiota Alpha-Diversity in COVID-19 Patients with Type 2 Diabetes: The Role of SARS-CoV-2 Variants and Antibiotic Treatment. Pharmaceuticals.

[74] Petakh P., Griga V., Mohammed I.B., Loshak K., Poliak I., Kamyshnyiy A. (2022). Effects of Metformin, Insulin on Hematological Parameters of COVID-19 Patients with Type 2 Diabetes. Med. Arch..

[75] Halabitska I., Petakh P., Lushchak O., Kamyshna I., Oksenych V., Kamyshnyi O. (2024). Metformin in Antiviral Therapy: Evidence and Perspectives. Viruses.

[76] Perazza F., Leoni L., Colosimo S., Musio A., Bocedi G., D’avino M., Agnelli G., Nicastri A., Rossetti C., Sacilotto F. (2024). Metformin and the Liver: Unlocking the Full Therapeutic Potential. Metabolites.

[77] Buchynskyi M., Oksenych V., Kamyshna I., Vorobets I., Halabitska I., Kamyshnyi O. (2024). Modulatory Roles of AHR, FFAR2, FXR, and TGR5 Gene Expression in Metabolic-Associated Fatty Liver Disease and COVID-19 Outcomes. Viruses.

[78] Buchynskyi M., Oksenych V., Kamyshna I., Budarna O., Halabitska I., Petakh P., Kamyshnyi O. (2024). Genomic Insight into COVID-19 Severity in MAFLD Patients: A Single-Center Prospective Cohort Study. Front. Genet..

[79] Bataller R., North K.E., Brenner D.A. (2003). Genetic Polymorphisms and the Progression of Liver Fibrosis: A Critical Appraisal. Hepatology.

[80] Abdelmonem B.H., Abdelaal N.M., Anwer E.K.E., Rashwan A.A., Hussein M.A., Ahmed Y.F., Khashana R., Hanna M.M., Abdelnaser A. (2024). Decoding the Role of CYP450 Enzymes in Metabolism and Disease: A Comprehensive Review. Biomedicines.

[81] Belenichev I., Popazova O., Bukhtiyarova N., Savchenko D., Oksenych V., Kamyshnyi O. (2024). Modulating Nitric Oxide: Implications for Cytotoxicity and Cytoprotection. Antioxidants.

[82] Gan C., Yuan Y., Shen H., Gao J., Kong X., Che Z., Guo Y., Wang H., Dong E., Xiao J. (2025). Liver Diseases: Epidemiology, Causes, Trends and Predictions. Signal Transduct. Target. Ther..

[83] Björnsson H.K., Björnsson E.S. (2022). Drug-Induced Liver Injury: Pathogenesis, Epidemiology, Clinical Features, and Practical Management. Eur. J. Intern. Med..

[84] Rehman S.T., Rehman H., Abid S. (2021). Impact of Coronavirus Disease 2019 on Prevention and Elimination Strategies for Hepatitis B and Hepatitis C. World J. Hepatol..

[85] Jiménez-Mendoza J., Rivera-López F., González-Lara M., Valdez-Echeverría R., Castro-Narro G., Tore A., Uscanga-Domínguez L., Moctezuma-Velázquez C. (2022). Seroprevalence of Hepatitis B and C Viruses in Moderate and Severe COVID-19 Inpatients: A Cross-Sectional Study at a Referral Center in Mexico. Ann. Hepatol..

[86] He Y.F., Jiang Z.G., Wu N., Bian N., Ren J.L. (2022). Correlation between COVID-19 and Hepatitis B: A Systematic Review. World J. Gastroenterol..

[87] Elsharkawy A., Samir R., Abdallah M., Hassany M., El-Kassas M. (2023). The implications of the COVID-19 pandemic on hepatitis B and C elimination programs in Egypt: Current situation and future perspective. Egypt. Liver J..

[88] Petakh P., Isevych V., Kamyshnyi A., Oksenych V. (2022). Weil’s Disease—Immunopathogenesis, Multiple Organ Failure, and Potential Role of Gut Microbiota. Biomolecules.

[89] Weiskirchen R. (2015). Hepatoprotective and Anti-fibrotic Agents: It’s Time to Take the Next Step. Front. Pharmacol..

[90] Jadhav P.A., Thomas A.B., Pathan M.K., Chaudhari S.Y., Wavhale R.D., Chitlange S.S. (2025). Unlocking the therapeutic potential of unexplored phytocompounds as hepatoprotective agents through integration of network pharmacology and in-silico analysis. Sci. Rep..

[91] Halabitska I., Babinets L., Oksenych V., Kamyshnyi O. (2024). Diabetes and Osteoarthritis: Exploring the Interactions and Therapeutic Implications of Insulin, Metformin, and GLP-1-Based Interventions. Biomedicines.

[92] Halabitska I., Petakh P., Kamyshna I., Oksenych V., Kainov D.E., Kamyshnyi O. (2024). The interplay of gut microbiota, obesity, and depression: Insights and interventions. Cell. Mol. Life Sci..

[93] Powell-Wiley T.M., Poirier P., Burke L.E., Després J.-P., Gordon-Larsen P., Lavie C.J., Lear S.A., Ndumele C.E., Neeland I.J., Sanders P. (2021). Obesity and Cardiovascular Disease: A Scientific Statement from the American Heart Association. Circulation.

[94] Leon B.M., Maddox T.M. (2015). Diabetes and cardiovascular disease: Epidemiology, biological mechanisms, treatment recommendations and future research. World J. Diabetes.

[95] Dzhuryak V., Sydorchuk L., Sydorchuk A., Kamyshnyi O., Kshanovska A., Levytska S., Knut R., Sheremet M., Ivashchuk M., Petrynych O. (2020). The cytochrome 11B2 aldosterone synthase gene CYP11B2 (RS1799998) polymorphism associates with chronic kidney disease in hypertensive patients. Biointerface Res. Appl. Chem..

[96] Bernhard J., Galli L., Speidl W.S., Krychtiuk K.A. (2025). Cardiovascular Risk Reduction in Metabolic Dysfunction-Associated Steatotic Liver Disease and Metabolic Dysfunction-Associated Steatohepatitis. Curr. Cardiol. Rep..

[97] Gutiérrez-Cuevas J., Santos A., Armendariz-Borunda J. (2021). Pathophysiological Molecular Mechanisms of Obesity: A Link between MAFLD and NASH with Cardiovascular Diseases. Int. J. Mol. Sci..

[98] Țenea-Cojan Ș.-T., Dinescu V.-C., Gheorman V., Dragne I.-G., Gheorman V., Forțofoiu M.-C., Fortofoiu M., Dobrinescu A.G. (2025). Exploring Multidisciplinary Approaches to Comorbid Psychiatric and Medical Disorders: A Scoping Review. Life.

[99] Rayman G., Akpan A., Cowie M., Evans R., Patel M., Posporelis S., Walsh K. (2022). Managing patients with comorbidities: Future models of care. Future Healthc. J..

[100] Kloock S., Ziegler C.G., Dischinger U. (2023). Obesity and its comorbidities, current treatment options and future perspectives: Challenging bariatric surgery?. Pharmacol. Ther..

